# Reversion of the *Arabidopsis* *rpn12a-1* exon-trap mutation by an intragenic suppressor that weakens the chimeric 5’ splice site

**DOI:** 10.12688/f1000research.2-60.v2

**Published:** 2013-06-18

**Authors:** Jasmina Kurepa, Yan Li, Jan A Smalle

**Affiliations:** 1 Plant Physiology, Biochemistry, Molecular Biology Program, Department of Plant and Soil Sciences, University of Kentucky, Kentucky, 40546, USA

## Abstract

**Background:** In the *Arabidopsis* 26S proteasome mutant *rpn12a-1*, an exon-trap T-DNA is inserted 531 base pairs downstream of the *RPN12a* STOP codon. We have previously shown that this insertion activates a STOP codon-associated latent 5' splice site that competes with the polyadenylation signal during processing of the pre-mRNA. As a result of this dual input from splicing and polyadenylation in the *rpn12a-1* mutant, two *RPN12a* transcripts are produced and they encode the wild-type RPN12a and a chimeric RPN12a-NPTII protein. Both proteins form complexes with other proteasome subunits leading to the formation of wild-type and mutant proteasome versions. The net result of this heterogeneity of proteasome particles is a reduction of total cellular proteasome activity. One of the consequences of reduced proteasomal activity is decreased sensitivity to the major plant hormone cytokinin.

**Methods:** We performed ethyl methanesulfonate mutagenesis of *rpn12a-1* and isolated revertants with wild-type cytokinin sensitivity.

**Results:** We describe the isolation and analyses of suppressor of *rpn12a-1* ( *sor1*). The *sor1* mutation is intragenic and located at the fifth position of the chimeric intron. This mutation weakens the activated 5' splice site associated with the STOP codon and tilts the processing of the *RPN12a* mRNA back towards polyadenylation.

**Conclusions:** These results validate our earlier interpretation of the unusual nature of the *rpn12a-1* mutation. Furthermore, the data show that optimal 26S proteasome activity requires RPN12a accumulation beyond a critical threshold. Finally, this finding reinforces our previous conclusion that proteasome function is critical for the cytokinin-dependent regulation of plant growth.

## Introduction

The 26S proteasome (26SP) is a multisubunit protease responsible for the degradation of proteins that are covalently labeled with a polyubiquitin (Ub) chain via the combined action of Ub activating enzymes, Ub conjugating enzymes and Ub ligases ^1^. The 26SP is localized in the cytosol and the nucleus, and it degrades proteins involved in many signaling and metabolic pathways ^1, 2^. The 26SP is also essential for the destruction of misfolded proteins that are generated by mistranslations and during stress ^2– 4^.

Studies with proteasome mutants in *Arabidopsis* have revealed that the 26SP is required for both male and female gametogenesis, confirming its essential role in plant growth and development ^2, 5, 6^. Partial loss-of-function mutants, on the other hand, have been indispensable for uncovering pathways in which key components are regulated by proteasome-dependent degradation ^7– 13^.

The *rpn12a-1* mutant, which carries an insertion in the *RPN12a* gene (At1g64520) encoding the regulatory particle non-ATPase subunit (RPN) 12a, was isolated from a collection of exon-trap lines ^14, 15^. These lines were generated by transforming *Arabidopsis* plants (C24 accession) with a T-DNA construct that contains a promoterless neomycin phosphotransferase gene ( *NPTII*) without a starting methionine which is preceded by a 3´ splice site of the first intron of the apurinic endonuclease ( *APR*) ^14^. Kanamycin-resistant exon-trap lines are therefore predicted to have the *APR-NPTII* construct inserted downstream of an active promoter either in frame with the coding region or in a position that allows the formation of a novel, chimeric intron. The *rpn12a-1* mutation is unusual because the T-DNA is inserted downstream of the *RPN12a* gene, and both the full-length *RPN12a* cDNA and a chimeric *RPN12a-NPTII* cDNA are produced ^15^. This suggested that two types of *cis* signals involved in the pre-mRNA processing of *RPN12a* are competing. Because the wild-type transcript is produced in the mutant and is stable enough to be detected using routine RNA analytical procedures, the poly(A) signal of the *RPN12a* gene must be intact and active. On the other hand, since a chimeric *RPN12a-NPTII* transcript is also produced, the 3´ splice site of the inserted T-DNA must have recruited a latent 5´ splice site in the *RPN12a* gene. We have previously shown that this predicted latent 5´ splice site is STOP codon-associated, and that the pre-mRNA splicing of the chimeric intron leads to the production of the fusion mRNA ^15^. As a result of the action of these two opposing pre-mRNA processing mechanisms, one part of the mRNA species transcribed from the mutant *RPN12a* gene is translated into a functional RPN12a protein, and the other is translated into a chimeric RPN12a-NPTII fusion protein. Because both RPN12a forms are incorporated into the 26SP, the total proteasome activity in these mutant seedlings is reduced, but not abolished ^15^.

The reduction of 26SP activity in *rpn12a-1* caused a pleiotropic phenotype, which included altered responses to cytokinins ^15^. Cytokinins are plant hormones that are essential for every aspect of growth and development ^16– 19^. For example, cytokinins control the development of meristems and vasculature, and play an important role in senescence and nutrient allocation ^19, 20^. To gain better insight into the cytokinin insensitivity of *rpn12a-1* seedlings, we screened for suppressor mutants that have a wild-type cytokinin growth response. Here we describe the intragenic *suppressor of rpn12a-1* ( *sor1*) that disrupts the latent 5´ STOP-associated splice site. *Sor1* reduced the expression of the *RPN12a-NPTII* fusion mRNA with a concomitant increase in *RPN12a* transcript level. As a result, RPN12a accumulation in *sor1* seedlings was identical to the wild-type and was accompanied by wild-type cytokinin sensitivity. These results validate our transcript processing interpretation of the *rpn12a-1* exon-trap effect and accentuate the importance of optimal RPN12a expression for cytokinin signaling.

## Materials and methods

### Plant material and growth conditions

The *Arabidopsis thaliana rpn12a-1* mutant in the C24 background was described by us previously ^15^. To grow plants on soil and in axenic cultures, seeds were surface-sterilized in 70% ethanol followed by 50% bleach and plated on MS/2 medium that contained half-strength MS salts (pH 5.7, Sigma, St. Louis, MO) and 1% (w/v) sucrose. The seeds were kept for 4 days in darkness at 4°C, and either plated on MS/2 or on soil (Miracle-Gro potting mix:Perlite at 1:1 ratio). Plants were grown in continuous light at 22°C.

### EMS mutagenesis and screening for *rpn12a-1* suppressors

The *rpn12a-1* seeds were pre-incubated in 1.0% KCl for 12 hours, and then mutagenized for 5 hours in 100 mM sodium phosphate buffer (pH 5) containing 5% DMSO and 80 mM ethyl methanesulfonate (EMS; Sigma-Aldrich, St. Louis, MO). Seeds were washed twice in 100 mM sodium thiosulphate and then twice in distilled water. Seeds were incubated and chilled in 0.1% agar and sown directly to soil. All the seeds in the M2 generation were pooled upon harvest, surface-sterilized and plated on the MS/2 medium containing 0.1 µM kinetin (6-furfurylaminopurine; obtained from Duchefa Biochemie by Gold Biotechnology, St. Louis, MO, USA). The putative suppressor mutants were transferred from the selection medium onto MS/2 medium to allow recovery, and were then transferred to soil.

### Phenotypic analyses of *sor1*

Cytokinin treatments were as previously described ^15^. For fresh weight analyses, seedlings were germinated and grown on kanamycin-containing media, and their weight was measured in pools of 5 seedlings after 24 days of growth. Kanamycin monosulfate was obtained from Gold Biotechnology.

### Expression analyses

Total RNA was isolated from *Arabidopsis* seedlings grown in liquid Gamborg’s B5 medium with 1% sucrose (pH 5.7) using TRIzol reagent (Invitrogen, Carlsbad, CA, USA). The iScript kit (BioRad, Hercules, CA, USA) and 1 µg of TURBO DNAse (Ambion, Austin, TX, USA) pre-treated total RNA was used for the synthesis of the first-strand cDNA. For the RT-PCR experiments, the primers used for the amplification of wild-type cDNA fragment (306 bp in length) were F1: 5´-GGGTGCCTATAACCGTGTGTTGAGTGCTAG-3´ and R1: 5´-ATACGCTCCAGCTCTCTGGCGTAGCTTAGA-3´. The *RPN12A-NPTII* fusion transcript fragment was amplified with F1 and *NPTII* primer R2: 5´-CCCCTGCGCTGACAGCCCGGAACA-3´. *PBA1* (At4g31300) was amplified using forward and reverse primers that contained the first and last 25 bp of the cDNA. The primer set used to amplify the *Arabidopsis* elongation factor 1-α (EF-1-α; At5g60390) was previously described ^9^. For the quantitative RT-PCR (qPCR), primers were designed using RealTime PCR tool (Integrated DNA Technologies, Coralville, IA, USA). The *RPN12a* fragment was amplified using qRPN12a F 5´-AGTTCGAGAGATTCAAGGCG-3´ and qRPN12a R 5´-TCCTCGGTTTTGACGCTTAG-3´ primers. The *RPT2a* (At4g29040) fragment was amplified by using 5´-AATCGGCAAGGAGATCGGAAACCT-3´ and 5´-TCGCCACAAACTCTTCCTCCATCA-3´ as F and R primers, respectively. Previous analyses ^21^ have shown that the best reference gene for the qPCR analyses of proteasome mutants is ACT2 (At3g18780)23. The qPCR assays were done as previously described ^22^.

For immunoblotting analyses, total proteins were isolated, separated and transferred to nitrocellulose membranes as described ^15^. Rabbit polyclonal anti-RPN12a and anti-PBA1 antibodies (used at 1:1000 dilution) were purchased from Enzo Life Sciences (Plymouth Meeting, PA, USA). The rabbit, polyclonal anti-NPTII antibodies (used at 1:1000) were obtained from Abcam (Cambridge, MA, USA).

### Analyses of the *sor1* mutation

Genomic DNA fragments from *rpn12a-1* and *sor1* were amplified using F1 and R2 primers and sequenced using dye-termination chemistry (Perkin-Elmer, Foster City, CA, USA) at Advanced Genetic Technologies Center (AGTC, KY, USA). Sequences were analyzed using Vector NTI Suite (Invitrogen, Carlsbad, CA).

## Results and discussion

### Isolation of an intragenic *rpn12a-1* suppressor

To obtain *rpn12a-1* suppressors, we mutagenized seeds with EMS and plated ~50,000 M2 seeds on a medium with 0.1 µM of the cytokinin kinetin. Because wild-type plants grown on 0.1 µM kinetin are chlorotic and smaller compared to *rpn12a-1* ^15^, we selected 14-day-old M2 seedlings which were pale green and small as putative suppressors. These putative suppressors were first transferred to cytokinin-free media to recover, and subsequently to soil for self-pollination. We isolated several classes of candidate mutants with varying degrees in *rpn12a-1* suppression. However, only one of these mutant lines displayed a near-complete reversion to the wild-type phenotype. Here we describe the molecular analyses of this line that we named *suppressor of rpn12a-1 1* ( *sor1*).

**Figure 1.**
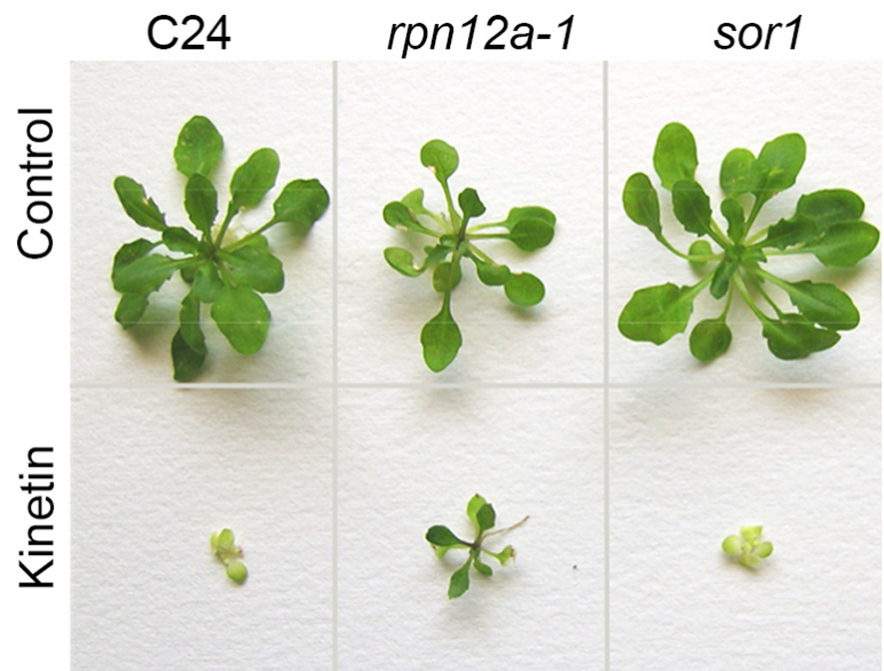
Decreased sensitivity of *rpn12-1* to cytokinins is restored by the *sor1* mutation. Plants were grown for three weeks on MS/2 media (control) or MS/2 media containing 0.2 µM kinetin in continuous light. Representative seedlings are shown.

Analyses of the M3 generation showed that in *suppressor of rpn12a-1 1* ( *sor1*), all visible phenotypes of *rpn12a-1* were reverted back to the wild-type ( Figure 1). For example, the *rpn12a-1* mutant has a smaller rosette than the wild-type and a reduced leaf initiation rate ^15^. The *sor1* plants had a leaf number and rosette size similar to the C24 wild-type plants ( Figure 1). The *sor1* mutant plants also displayed wild-type sensitivity to cytokinin. After three weeks of growth on a medium with 0.2 µM kinetin, both wild-type and *sor1* seedlings were chlorotic and their growth was severely inhibited, while the *rpn12a-1* seedlings were green and larger ( Figure 1).

**Figure 2.**
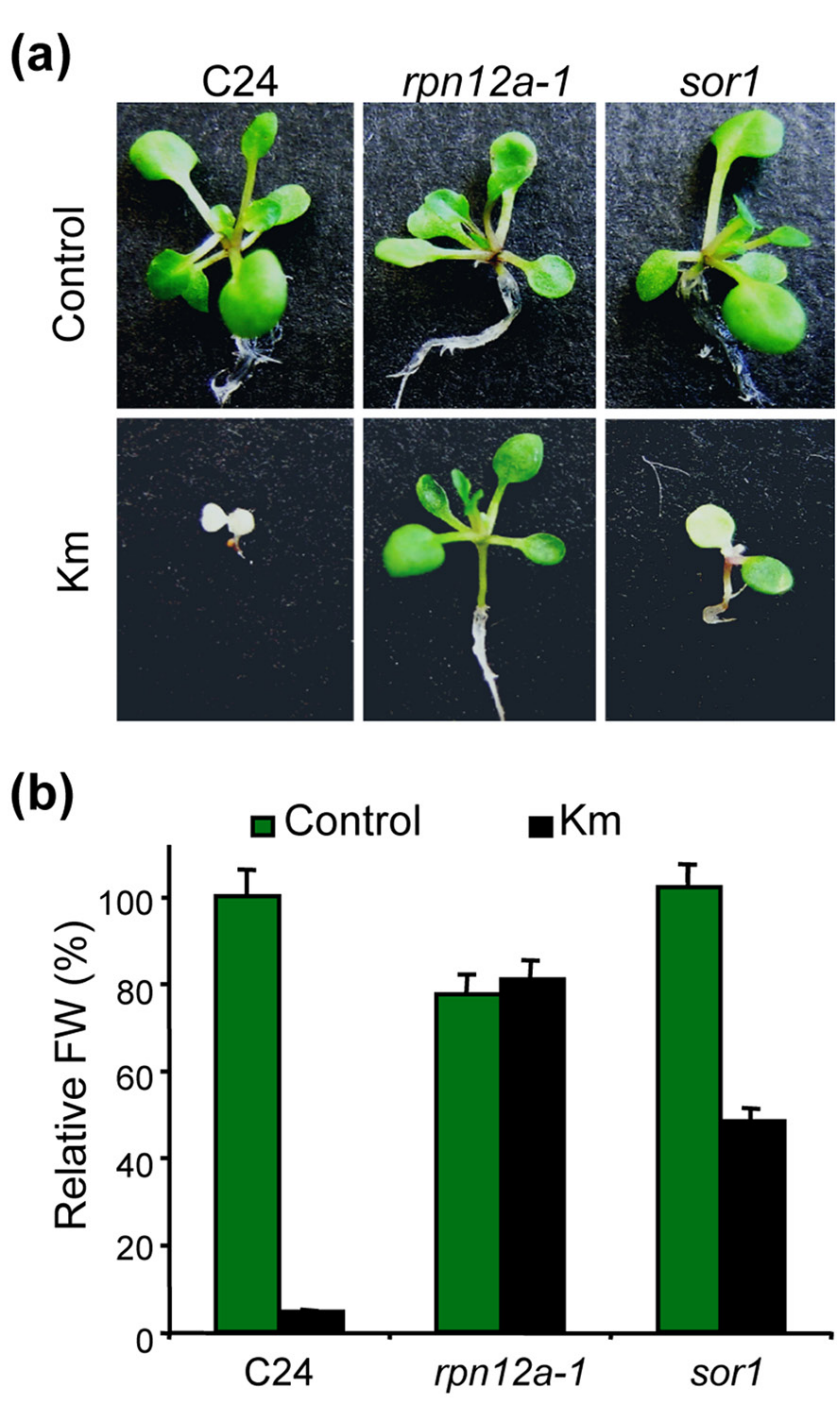
The *sor1* mutation leads to a partial loss of kanamycin resistance. ( **a**) Wild-type (C24), *rpn12a-1* and *sor1* seeds were sown and grown on MS/2 media containing 35 µg/ml kanamycin (Km). Representative plants were photographed after two weeks of growth. ( **b**) Fresh weight (FW) of seedlings grown on Km media was measured after two weeks of growth. FW of the wild-type plants grown on control MS/2 media was calculated as 100%. Seedlings were measured in pools of five, and mean ± SD is presented (n≥7).

**Figure 3.**
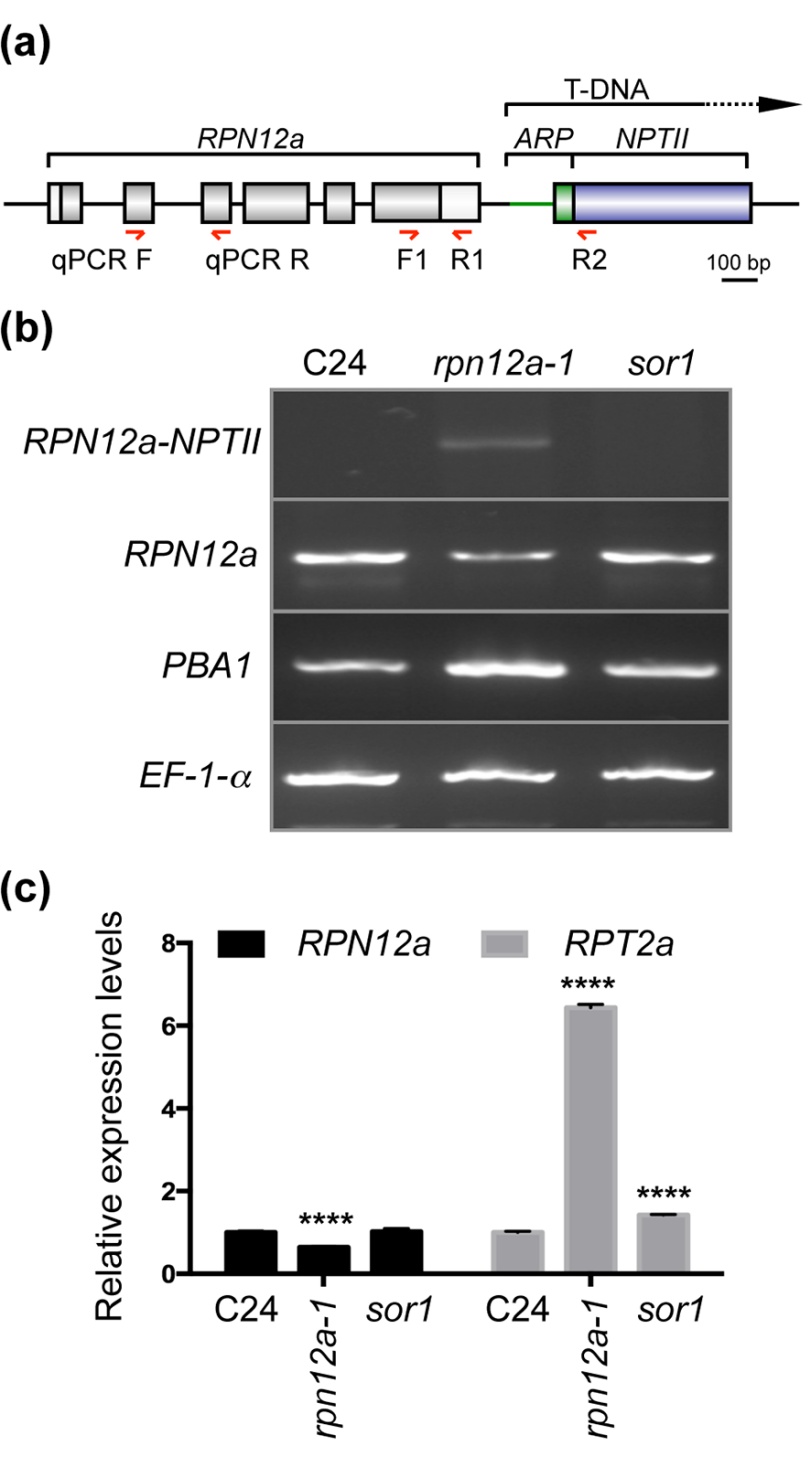
The *sor1* mutation reduces the expression of the *RPN12a-NPTII* fusion transcript. ( **a**) Simplified schematic representation of the *RPN12a* gene and the inserted T-DNA in the *rpn12a-1* mutant ^15^. The T-DNA contains the first intron and second exon of the apurinic endonuclease gene ( *ARP*) fused in frame to the neomycin phosphotransferase II ( *NPTII*) coding region. Exons are represented by gray boxes and introns as lines. Positions of the forward (F1) and reverse (R1 and R2) primers used for the RT-PCR and qRPN12a F and R primers used for qPCR are indicated. ( **b**) Total RNA was extracted, reverse transcribed and used to amplify the *RPN12a-NPTII* (42 cycles) and wild-type *RPN12a* transcripts (35 cycles). The primers used for the reaction are indicated. Proteasome β subunit 1 ( *PBA1)* elongation factor 1-α ( *EF-1-α*) are controls. ( **c**) Quantitative real-time RT-PCR analyses of *RPN12a* and *RPT2a* levels in C24, *rpn12a-1* and *sor1* seedlings. The reference gene was *ACT2*. The transcript levels in C24 were assigned the value of 1. The data represent average relative quantity (RQ) values of three replicates, and the bars denote the RQMin to RQMax. The difference in transcript levels between C24 and the mutants is marked (****, *P* < 0.0001; ANOVA with Bonferroni multiple comparison test).

Next, we analyzed the kanamycin (Km) resistance of the *sor1* mutant line. The Km resistance of the *rpn12a-1* mutant is completely linked to the proteasome-related phenotypes and thus, all the progeny of a plant homozygous for the *rpn12a-1* mutation should be Km resistant. All *sor1* seedlings were indeed resistant to Km, but the levels of resistance were significantly lower compared to *rpn12a-1* ( Figure 2). While Km did not affect the growth of *rpn12a-1* seedlings, both root and shoot growth of *sor1* were partially inhibited ( Figure 2). We did not observe any attenuation of Km resistance over several generations, a phenomenon that has been documented for a number of T-DNA insertion mutant collections ^23^ (see also the Salk Institute Genomic Analysis Laboratory Arabidopsis sequence indexed T-DNA insertion Project FAQ). An explanation for the change in Km tolerance in *sor1* is that the mutation affects the expression of the *NPTII* gene which is an integral part of the exon-trap ( Figure 3a and Babiychuk *et al.* 1997 ^14^). When the *sor1* mutant was outcrossed to the C24 wild type, none of the plants of the F2 population displayed an *rpn12a-1* phenotype, indicating that *sor1* is intragenic and tightly linked with the *rpn12a-1* mutation.

### *sor1* suppresses the accumulation of the *RPN12a-NPTII* fusion transcript

To obtain further insight into the nature of the *sor1* mutation, we analyzed the expression of the *RPN12a* gene and the accumulation of the RPN12a protein. RT-PCR analyses showed that in *sor1*, the *RPN12aNPTII* fusion transcript was not detectable and that the *RPN12a* cDNA level was comparable to the wild type ( Figure 3b). Quantitative RT-PCR (qPCR) analyses confirmed that there was no statistically significant difference between *RPN12a* levels in *sor1* and the wild type ( Figure 3c). The fusion transcript, which was not detected in the C24 line, was present in the *sor1* plants at a ratio of 1:15,000 compared to the *rpn12a-1* mutant (relative transcript levels were calculated to be 1.0 ± 1.2 and 15, 856 ± 542 for *sor1* and *rpn12a-1*, respectively).

Reductions in proteasome activity typically lead to the activation of a feedback mechanism that induces the transcription of proteasome subunit genes. This mechanism is operational in all eukaryotes, including yeasts, *Drosophila*, mammals and plants ^7, 24– 28^. Due to this global feedback up-regulation of 26SP subunit genes, the 20S proteasome subunit β1 ( *PBA1*) and 26SP regulatory particle subunit *RPT2a* transcripts were more abundant in *rpn12a-1* compared to the wild type ( Figure 3b and 3c). RT-PCR analyses suggested and qPCR analyses confirmed that the proteasome subunit transcript levels in *sor1* were reduced compared to *rpn12a-1*, but still increased compared to the wild-type ( Figure 3b and 3c), indicating that the *sor1* mutation did not lead to a complete suppression of the *rpn12a-1* mutation.

**Figure 4.**
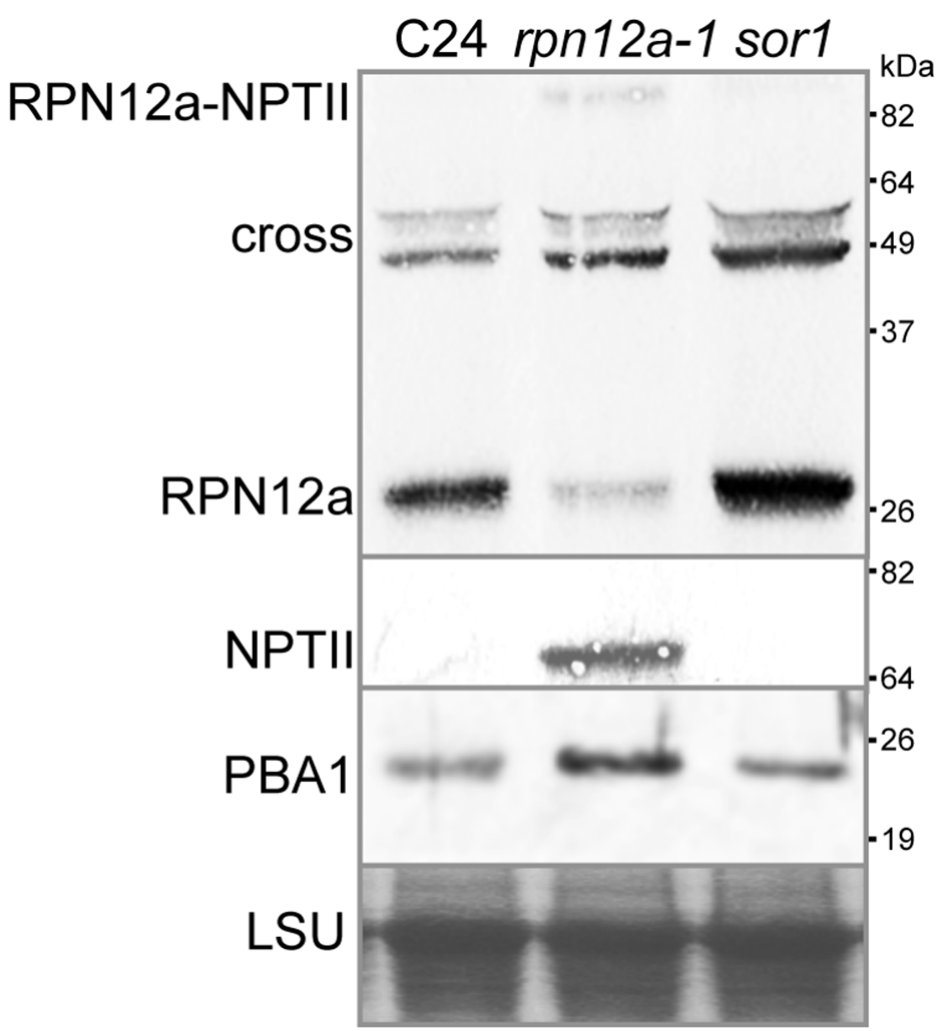
The RPN12a-NPTII fusion protein is absent in the *sor1* mutant. Total protein was isolated from two-week-old wild-type (C24), *rpn12a-1* and *sor1* seedlings and used for immunoblotting analyses with RPN12, NPT and PBA1 antisera. In addition to the RPN12a and RPN12a-NPTII fusion proteins, the anti-RPN12 sera also recognized two proteins (cross) that are not related to RPN12a. Ponceau S-stained membrane showing the large RuBisCO subunit (LSU) is presented as a loading control. The size of the proteins used as molecular mass standards is shown on the right-hand side.

Immunoblotting analyses using anti-RPN12a antibodies showed that the *sor1* mutant does not accumulate the RPN12a-NPTII fusion protein ( Figure 4). The RPN12a abundance in *sor1* was increased compared to *rpn12a-1* and similar to the wild-type. We were also unable to detect the RPN12a-NPTII fusion in *sor1* by using anti-NPTII antisera ( Figure 4). In the *rpn12a-1* mutant, a fraction of the assembled 26SP contains the fusion protein leading to a decrease in total cellular 26SP activity and a compensatory increase in the expression of proteasome subunit genes ^15, 28^. In the *sor1* mutant, with no or little fusion protein, 26SP function is expected to be restored back to the wild-type level. Indeed, immunoblotting analyses with the anti-PBA1 antibodies showed that the abundance of the 20S proteasome subunit PBA1 in *sor1* seedlings was comparable to that of the wild-type, indicating that proteasome activity was restored to optimal levels and that feedback up-regulation of proteasome subunit genes was halted ( Figure 4).

Taking into account both the result of the Km resistance tests ( Figure 2) and the expression data ( Figure 3 and Figure 4), we concluded that the *sor1* mutation strongly but incompletely suppresses the formation of the *RPN12a-NPTII* fusion transcript which was sufficient to restore 26SP function back to the wild-type level.

### *sor1* weakens the STOP codon-associated 5´ splice site in *rpn12a-1*

**Figure 5.**
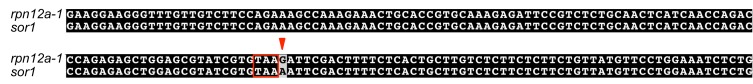
Sequence alignment of the *RPN12a* gene (At1g64520) in *rpn12a-1* and *sor1*. Genomic DNA fragment was amplified using F1 and R2 primers (presented in Figure 3), sequenced and the sequence was aligned using Vector NTI suite. Alignment of the region starting with base pair 1615 and ending with base pare 1804 of the annotated *RPN12a* gene is presented using BoxShade 3.2. The red arrowhead points to the *sor1* mutation and the *RPN12a* STOP codon is boxed in red.

To find the mutation that causes the *sor1* phenotype, we amplified and compared the sequences of the *RPN12a-NPTII* chimeric gene from *sor1* and *rpn12a-1*. No mutations were found in *NPTII*, indicating that the loss of Km resistance and NPTII abundance was not caused by any disruption of the *NPTII* coding region. We also did not detect any changes in the *RPN12a* coding region, but did find a single nucleotide change immediately downstream of the *RPN12a* STOP codon ( Figure 5). Sequencing of the entire region between *RPN12a* and *NPTII* did not reveal any additional mutations, confirming that the *RPN12a* STOP codon-associated G to A mutation was indeed *sor1*.

**Figure 6.**
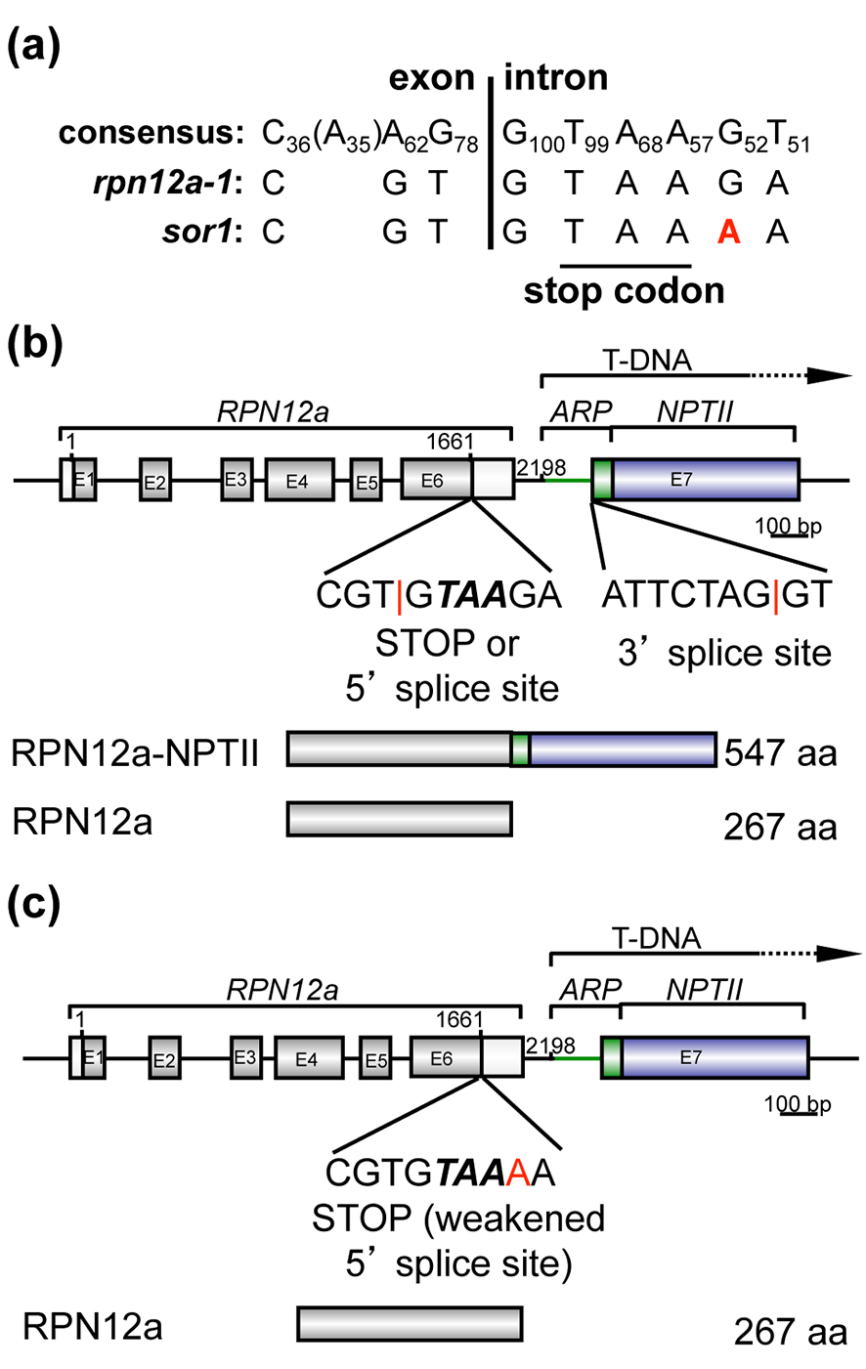
The *sor1* mutation weakens the cryptic 5´ splice site that includes the STOP codon of the *RPN12a* gene. ( **a**) Sequence alignment of the terminal exonic tetranucleotides and proximal intronic hexanucleotides of the *Arabidopsis* consensus sequence ^29^, and *rpn12a-1* and *sor1* sequences surrounding the STOP codon. Numbers next to the nucleotides of the consensus sequence refer to the frequency (%) for the noted nucleotide to be found at a given position. ( **b**), ( **c**) Schematic representations of splicing types in *rpn12a-1* ( **b**) and *sor1* ( **c**). aa, amino acids.

To analyze how this G-to-A substitution leads to reversion of the *rpn12a-1* phenotype, we manually compared the consensus sequence for 5´ splice sites in *Arabidopsis* ^29^ with the sequence of the exon/intron junction that precedes the *RPN12a* STOP codon in *rpn12a-1* and *sor1* ( Figure 6a). The alignment revealed that both the intron and exon residues adjoining the splice junction of the mutants match the consensus well. Interestingly, the *sor1* mutation changes a consensus G at the fifth position of the intron into an A, thus weakening the 5´ splice site of the chimeric intron. The G at the position +5 is thought to be required for efficient binding of U1snRPN ^29^. Reduced splicing of the chimeric intron between the *RPN12a* and *NPTII* coding regions is predicted to lead to a reduced accumulation of the *RPN12a-NPTII* transcript and protein ( Figure 6b and 6c). The combination of reduced intron splicing and unaffected 3´ end processing is therefore predicted to lead to a dramatic shift in favor of the formation of the wild-type *RPN12a* transcript, and thus to the accumulation of the RPN12a protein back to the wild-type level, which is what we observed in *sor1* seedlings.

## Conclusions

Collectively, the results shown here validate our earlier interpretation of the effects of the *rpn12a-1* mutation on *RPN12a* expression and 26SP function ^15^. In the original study, we proposed that the partial loss of 26SP function in *rpn12a-1* seedlings is caused by the competition between *RPN12a* and *RPN12a-NPT-II* transcript processing that leads to a decrease of RPN12a protein levels and thus, to a decrease in the abundance of wild-type 26SP particles ^15^. Our finding that suppression of *RPN12a-NPTII* accumulation was sufficient to restore RPN12a accumulation and reverse the plant development and cytokinin sensitivity back to the wild-type level validates the proposed interpretation and accentuates the importance of optimal 26SP abundance for *Arabidopsis* growth and cytokinin regulation ^1, 2, 15, 22, 30^.
