# Supplementary material for: Reversion of the Arabidopsis rpn12a-1 exon-trap mutation by an intragenic suppressor that weakens the chimeric 5’ splice site
Source: F1000Res. 2013 Jun 18;2:60. Originally published 2013 Feb 25. [Version 2] doi: 10.12688/f1000research.2-60.v2 (PMC3829128; doi:10.12688/f1000research.2-60.v2)
Supplement: Peer review history

# Peer review: Referee response for version 2

**Published:** 2013-07-01

**Authors:** Patrick Masson

**Affiliations:** 1 Department of Genetics, University of Wisconsin-Madison, Madison, WI, USA

This revised manuscript includes both a better description of the mutagenesis and screening strategy that led to the isolation of *sor1* and a new experiment quantifying transcript levels. This addresses two of my initial questions. A transgenic-rescue experiment aimed at verifying that the mutation detected immediately downstream of the RPN12a STOP codon truly corresponds to *sor1* has not been carried out. Although I still believe this would be useful to verify this conclusion, I also think that all other data lead us to believe in the proposed model.

I have read this submission. I believe that I have an appropriate level of expertise to confirm that it is of an acceptable scientific standard.

# Peer review: Referee response for version 2

**Published:** 2013-06-25

**Authors:** Vitaly Citovsky

**Affiliations:** 1 Department of Biochemistry and Cell Biology, State University of New York at Stony Brook, Stony Brook, NY, USA

The revised paper addresses my suggestion and strengthens the manuscript.

I have read this submission. I believe that I have an appropriate level of expertise to confirm that it is of an acceptable scientific standard.

# Peer review: Referee response for version 1

**Published:** 2013-03-06

**Authors:** Patrick Masson

**Affiliations:** 1 Department of Genetics, University of Wisconsin-Madison, Madison, WI, USA

This manuscript nicely documents the molecular basis for an intragenic suppressor of the *rpn12a-1* exon-trap mutation of *Arabidopsis*, which weakens a chimeric 5’ splice site that fuses the *RPN12* open reading frame to the *NPTII* coding region in the original mutation. These authors had previously shown that the T-DNA insertion of *rpn12a-1* results in a competition between 3’ splicing of *RPN12A* RNA (using a donor splice site that overlaps with the stop codon and the acceptor splice site upstream of its NPTII coding region of the T-DNA) and its normal polyadenylation. They had suggested that a fraction of the mutant transcripts encoded a non-functional RPN12A-NPTII fusion protein that, upon insertion into the proteasome, altered its activity. Hence, in the original mutant, overall altered proteasome activity resulted in pleiotropic phenotypes associated with cytokinin resistance compared to wild type. In this suppressor line, a point mutation 5 nucleotides within the cryptic intron altered this competing splicing, thereby restoring more efficient polyadenylation and production of enough functional RPN12a protein to restore fully functional proteasome activity. Hence, this analysis confirms the initial interpretation of the source of phenotypes associated with *rpn12a-1*, and documents an interesting example of alteration through mutation of a balance between 3’ splicing and polyadenylation of a precursor RNA.

The design of this work, protocols and results are well presented and justify the conclusions. However, I had a few minor questions on this work:

- How many suppressors were identified in this analysis? Were other intragenic suppressors identified?
- Considering the information provided here, one would suspect that sor1 is a dominant mutation. Is it? If it is, has an experiment been carried out to show that a transgenic copy of the suppressed rpn12a-1 sor rescues the cytokinin-resistance phenotype of rpn12a-1?
- Analysis of kanamycin resistance in wild type C24, rpn2a-1 and sor1 seedlings showed that sor1 retains a reasonably high level of resistance compared to wild type (Fig 2). Yet, the molecular characterization described in Figures 3 and 4 shows no evidence of NPTII transcript or protein being produced in this suppressor. Is this a problem of experimental sensitivity? A brief discussion of this observation should be included in the text.

I have read this submission. I believe that I have an appropriate level of expertise to confirm that it is of an acceptable scientific standard.

# Peer review: Referee response for version 1

**Published:** 2013-02-27

**Authors:** Vitaly Citovsky

**Affiliations:** 1 Department of Biochemistry and Cell Biology, State University of New York at Stony Brook, Stony Brook, NY, USA

This is a very nicely executed and clearly written work. The results are clear, and they support the authors’ conclusions and previously published hypotheses of proteasome involvement in cytokinin response. 

One potential enhancement would be to use qPCR to quantify the amount of transcripts, especially since these data represent one of the major findings of the paper.

I have read this submission. I believe that I have an appropriate level of expertise to confirm that it is of an acceptable scientific standard.
